# Hansen Solubility Parameters Applied to the Extraction of Phytochemicals

**DOI:** 10.3390/plants12163008

**Published:** 2023-08-21

**Authors:** Fábio Junior Moreira Novaes, Daliane Cláudia de Faria, Fabio Zamboni Ferraz, Francisco Radler de Aquino Neto

**Affiliations:** 1 Departamento de Química, Universidade Federal de Viçosa, Avenida Peter Henry Rolfs, s/n, Campus Universitário, Viçosa 36570-900, MG, Brazil; fabio.novaes@ufv.br (F.J.M.N.); daliane.faria@ufv.br (D.C.d.F.); fabio.ferraz@ufv.br (F.Z.F.); 2 Laboratório de Apoio ao Desenvolvimento Tecnológico (LADETEC/IQ-UFRJ), Instituto de Química, Universidade Federal do Rio de Janeiro, Rio de Janeiro 21941-598, RJ, Brazil

**Keywords:** predictive solubility model, group contribution method, solvent selection, alternative solvents, analytical green chemistry strategy

## Abstract

In many analytical chemical procedures, organic solvents are required to favour a better global yield upon the separation, extraction, or isolation of the target phytochemical analyte. The selection of extraction solvents is generally based on the solubility difference between target analytes and the undesired matrix components, as well as the overall extraction procedure cost and safety. Hansen Solubility Parameters are typically used for this purpose. They are based on the product of three coordinated forces (hydrogen bonds, dispersion, and dipolar forces) calculated for any substance to predict the miscibility of a compound in a pure solvent, in a mixture of solvents, or in non-solvent compounds, saving time and costs on method development based on a scientific understanding of chemical composition and intermolecular interactions. This review summarises how Hansen Solubility Parameters have been incorporated into the classical and emerging (or greener) extraction techniques of phytochemicals as an alternative to trial-and-error approaches, avoiding impractical experimental conditions and resulting in, for example, saving resources and avoiding unnecessary solvent wasting.

## 1. Introduction

Intermolecular forces are responsible for the physical state of matter and other physical–chemical properties, such as melting, boiling and flash points, viscosity, surface tension, capillarity, density, and solubility. Among neutral organic molecules, most of the interactions are based on London or van der Waals dispersion forces, dipole–dipole interactions, and hydrogen bonding. London or van der Waals dispersion forces are present in all substances, produced via the distortion of the electron cloud and instantaneous or induced formation of momentary dipoles. For atoms, the force is directly proportional to the number of electrons: the greater the number of electrons, the greater the momentary dispersion of the electronic cloud. For molecules, the force increases in intensity with increasing molecular mass (I_2_ > Br_2_ > Cl_2_ > F_2_) and the state of aggregation (pentane > methylbutane > dimethylpropane) associated with the contact surface between the molecules. Permanent dipole–dipole interactions come from the dipole moment and are the result of the difference in electronegativity between the atoms of an intramolecular chemical bond. The higher this difference, the greater the polarization and dipole–dipole force. Lastly, hydrogen bonding is a special type of intermolecular attraction between the hydrogen atom in a polar bond (particularly an H-F, H-O, or H-N bond) and an unshared electron pair of a small and electronegative ion or atom that is close (usually an F, O, or N atom) to a neighbouring molecule. This is because the hydrogen atom has only one electron, which is used in bond formation; therefore, the positive side of the bond dipole has the concentrated charge partially exposed. This positive charge is attracted by the negative charge of a small electronegative atom in a nearby molecule. Given that electron-poor hydrogen is so small, it can become very close to an electronegative atom and interact strongly with it [1,2]. Hydrogen bonding can also occur intramolecularly, reducing the effectiveness of forming intermolecular interactions. These three intermolecular interactions are usually explained individually and without correlation with each other. Together, they govern the properties mentioned above and can be estimated and used to predict the solubility of a given analyte in a pure solvent, in a mixture of solvents, or in non-solvents. This is one of the legacies of Charles Medom Hansen [2,3].

Dr. Hansen is an American scientist who was born in 1938 in Louisville City, Kentucky, USA. After his graduation (1961, Louisville) and master’s degree in chemical engineering (1962, Wisconsin), Hansen moved to Denmark to complete his Ph.D. at the Technical University of Denmark (1967) to study the properties of the solvents used throughout his Ph.D. [2,3,4,5]. His first finding was the formation of a polymeric film produced via the evaporation of solvents during paint drying, governed by surface phenomena, solvent vapour pressure, air velocity, and heat transfer. The diffusion of solvent molecules inside the film to the outside is the second active phenomenon, which depends on the concentration of the solvent and its retention in the film since it can be found in paints and varnishes, even for years after application [3,5].

The reasons for the retention of solvents were explored based on their solubility with the following paint components: polymers, pigments, and other solvents present. For his first approach, Hansen used the theories of Hildebrand and Scott [6,7], who used only the London dispersion forces and a “polar” interactions to describe the solubility of substances. In this context, Hildebrand and Scott studied only non-polar substances. Hansen perfected this approach to describe the solubility of solvents, polymers, and pigments used in his thesis, which involves polar molecules based on the parameters of the momentary dipoles (London, $\delta_{L}$), permanent dipoles ($\delta_{P}$), and hydrogen bonds ($\delta_{H}$) [3,8,9]. In this scenario, liquids with close values of solubility parameters ($\delta_{L}$, $\delta_{P}$_,_ and $\delta_{H}$) are miscible; thus, polymers and pigments dissolve in solvents with solubility parameters close to theirs. These conditions were described as Hansen Solubility Parameters (HSPs), which were initially applied to correlate and compare solubility and surface permeation.

The HSPs are still used in paint and coating formulations, such as cleaning materials that remove them from surfaces [10,11,12,13]. They are also used in the formulation, dissolution, and storage stability of drugs for humans [14,15,16] and in the development of pest control agents [17]. In the energy field, the HSPs have been applied in the delignification of sugar cane bagasse for ethanol production [18], the incorporation of polyoxymethylene ethers in diesel fuels to reduce particulate matter [19], and the investigation of better co-solvents for improving fuel quality [20], among other applications. In separation science, the solvent-membrane affinity is estimated to facilitate the permeation of solvents and the removal of target residues [21,22], to describe the adsorption separation mechanism between mobile phase–adsorbent–adsorbed material [23,24], to select the mobile phase [25], and to predict the retention of analytes in chromatographic separations [26]. They are also involved in many other applications.

The goals of this review are to describe the HSPs as screening predictive tools for solvent selection on the extraction of target phytochemicals, to describe them as alternatives to trial-and-error approaches, and to outline how they may be used to save resources and avoid unnecessary solvent waste. The economic and ecological variables of the solvent are also discussed to support the analyst’s decision. The approaches applied to reach these goals are outlined below and include the Soxhlet extraction of lipids, the recovery of phytochemicals from natural sources via solid–liquid extraction (SLE), microwave-assisted extraction (MAE), pressurised liquid extraction (PLE), and ultrasound-assisted extraction (UAE), among others.

## 2. Physical Chemistry Definition and Meaning of HSPs

Solubility parameters can be referred to as cohesion energies, that is, those responsible for keeping the molecules of a liquid together; therefore, it is appropriate to relate these energies to surface phenomena. The total cohesion energy ($E_{coh}$) can be measured experimentally by completely evaporating the solvent and breaking all cohesion bonds; thus, it can be considered equal to the vaporisation energy (Equation (1)). For aliphatic hydrocarbons, the momentary dipole is the only existing interaction, allowing the matching of the vaporisation energy with the momentary cohesion energy ($E_{L}$), governed by London forces, as shown in Equation (1).

(1) $$E_{coh}=∆H_{vap}-RT=E_{L}$$

where $E_{coh}$, $∆H_{vap}$, R, and T are the cohesion energy, evaporation enthalpy of the pure substance, the general constant of the gases, and the temperature in Kelvin, of which its vapour should be considered ideal.

Previously, the polar contribution did not distinguish the permanent dipole from the hydrogen bond. This contribution was first described by Blanks [27], who investigated the homomorphic hydrocarbons of polar compounds in his study. By evaporating both molecules separately, Blanks [27] obtained their cohesion energies and subtracted the polar contribution of the $E_{coh}$ of the polar molecule from the $E_{coh}$ of the homomorphic hydrocarbon. However, Hansen decided to mathematically separate the two forces.

For Hansen, the permanent dipole–dipole interaction causes the second type of interaction, the polar cohesion energy ($E_{P}$). Mathematically, the contribution of permanent dipoles was originally Böttcher’s idea [28], presented as Equation (2), which Hansen used to determine the $E_{P}$. The equation was validated after Hansen obtained an adequate correlation coefficient between the results of Equation (2) and the experimental values derived from the vaporisation of different solvents that contained polar functional groups [28].

(2) $$E_{P}=\frac{-4\pi }{3}\times \frac{d\times {N_{A}}^{2}}{MM}\times \frac{\varepsilon -1}{2\varepsilon +{n_{D}}^{2}}\times \frac{{n_{D}}^{2}+2}{3}\times \mu^{2}$$

where d is the density, $N_{A}$ is the Avogadro number, MM is the molar mass, ε is the dielectric constant, $n_{D}$ is the refraction index, and μ is the dipole moment.

The third source of cohesive energy is the result of hydrogen bonding ($E_{H}$). The hydrogen bond parameter was used to approximate the energies of interactions not included in the two previous parameters and is present in molecules capable of donating or receiving hydrogen ions (alcohols, glycols, carboxylic acids, amino acids, and other hydrophilic compounds) and those only capable of receiving (ethers, tertiary amines, etc.) [3]. Based on infrared data, in which its OH–H interaction had a value close to 5000 cal mol^−1^, Hansen estimated that this value could compose the cohesive energy of the hydrogen bond, to be multiplied by *n*, the number of possible interactions for a given fragment (*k*) (Equation (3)) [28]. Again, reference curves (theoretical versus experimental values) were obtained by vaporising solvents that had functional groups capable of forming hydrogen bonding, with $E_{H}$ values calculated as follows:

(3) $$E_{H}=n\times aditive\;fragments\;(k)$$

There are other sources of cohesion energy, but to predict the similarity or difference in cohesion energy parameters, those presented were sufficient to confirm the attributions of the HSPs for different liquids. Thus, the basic equation that governs the HSPs, the total cohesion energy ($E_{Total}$), is the sum of the individual energies that compose it (Equation (4)).

(4) $$E_{Total}=E_{coh}=E_{L}+E_{P}+E_{H}$$

The cohesive energy increases proportionally with the molecular volume. Therefore, to facilitate the dimensioning of this measure, Hansen divided Equation (2) by the molar volume ($V_{M}$, cm^3^ mol^−1^) (Equation (5)), indicating that the energy density could be treated in terms of the solubility parameter (δ), according to Equation (6) derived from Hildebrand’s studies [6,7]. This modification yielded the classic formula for HSPs, in which the square of the total solubility is equal to the sum of the squares of the components *L*, *P*, and *H* of Hansen [28] (Equation (7)).

(5) $$\frac{E_{Total}}{V_{M}}=\frac{E_{L}}{V_{M}}+\frac{E_{P}}{V_{M}}+\frac{E_{H}}{V_{M}}$$

(6) $$\delta^{2}=\frac{E}{V_{M}}$$

(7) $$\delta_{Total}^{2}=\delta_{L}^{2}+\delta_{P}^{2}+\delta_{H}^{2}$$

**Figure 1:**
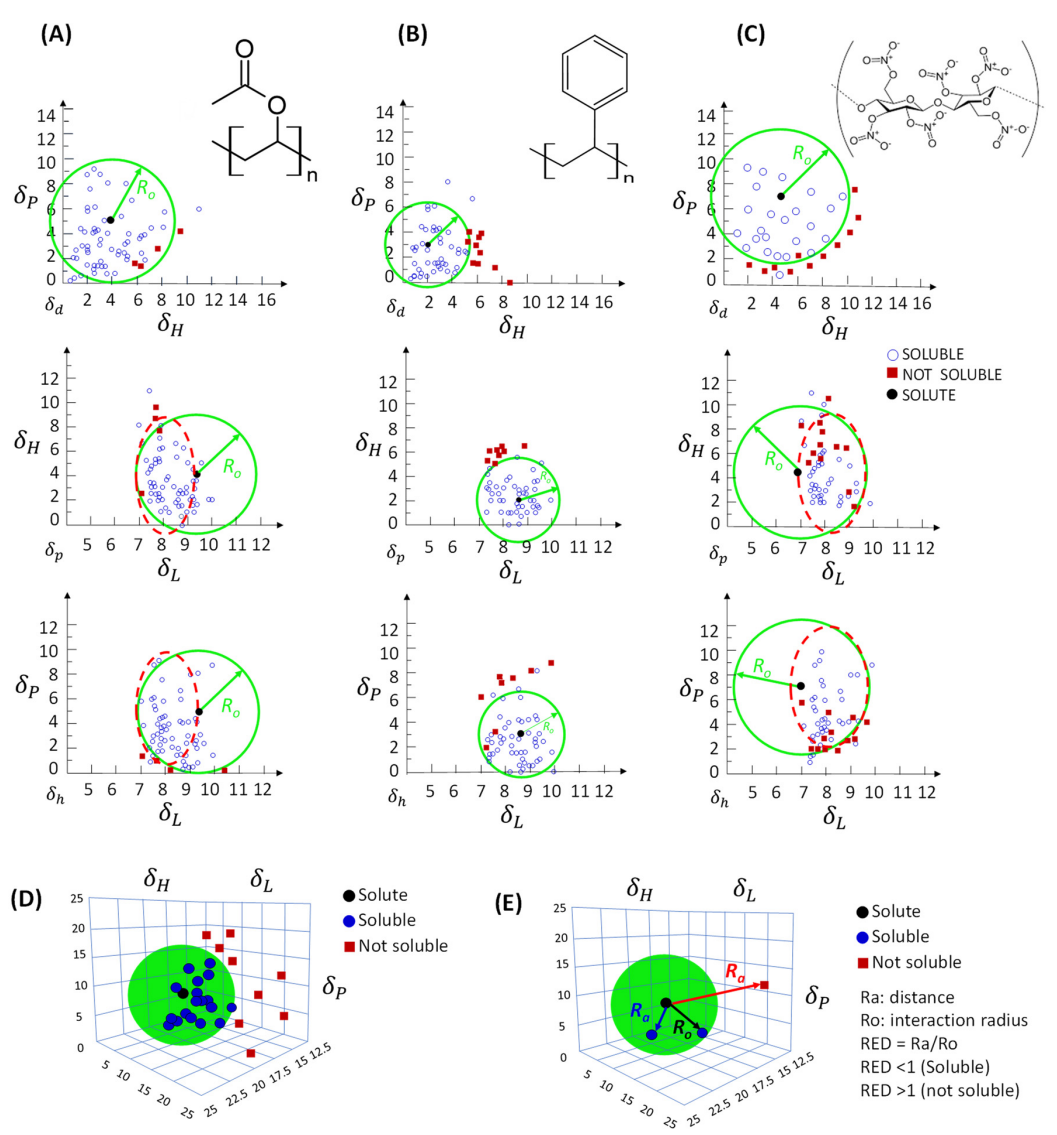
(**A**–**C**) Two- and (**D**,**E**) three-dimensional plots $\delta_{L}$, $\delta_{P}$, and $\delta_{H}$ of solute solubility sphere of Hansen (green circle) with its interaction radius (Ro), solute–solvent distance (Ra; Equation (8)), and their relative energy distances (RED; Equation (9)) [3,28].

The projection of the three HSPs onto the target solute locates it in two- and three-dimensional coordinate spaces $\delta_{L}$, $\delta_{P}$, and $\delta_{H}$ (Equation (7)). The same approach for solvents also results in their point locations around the target solute point. This space occupied by solvents, with the target solute in its centre, configures the spherical volume of solubility (or interaction) (Figure 1D,E). Thus, Hansen introduced the concept of solubility volume by producing a sphere (Figure 1D) that contained in its interior the solvents that experimentally dissolved the target solute, and in its exterior were solvents that were immiscible with it. 

The circles in Figure 1A–C were first represented for the construction of the model, which results in a three-dimensional spherical representation (Figure 1D). The closer to the target solute, the more similar would be the intermolecular forces of the solvent with the solute, and the greater the solubility between these species, that is, “like dissolves like”. Therefore, miscible substances are those close to each other in the graphic space, located inside the circle/sphere, while immiscible substances are far away, outside the circle/sphere of solubility (Figure 1). Both the region of the analyte and the solubility volumes have a certain degree of uncertainty (Figure 1) since the sphere is built by trial and error, which can be more statistically accurate with the increase in the number of solvents used for the construction of the graphic model [28]. It is also possible to have immiscible solvents within the solubility region as well as miscible solvents outside. These deviations usually occur with small molar volume solvents, such as methanol, and in the $\delta_{L}$ axis region due to the dispersive nature of London forces, resulting in ellipses and spheroids instead of circles and spheres (Figure 1: ellipses dashed in red) [28].

The distance between solute *i* and solvent *j* gives rise to Equation (8), which results in the distance of the solubility parameters of the two substances (Ra). This is the measure of how similar their intermolecular forces are: the lower the Ra between the two molecules, the greater the possibility of compatibility between their solubilities (Figure 1E) [3].

(8) $$Ra={∆\delta }_{ij}=\sqrt{4{\left(\delta_{L_{i}}-\delta_{L_{j}}\right)}^{2}+{\left(\delta_{P_{i}}-\delta_{P_{j}}\right)}^{2}+{\left(\delta_{H_{i}}-\delta_{H_{j}}\right)}^{2}}$$

The constant 4 of Equation (8) was determined empirically and considered convenient for adequately representing the solubility data as a sphere that encompasses miscible solvents [3]. The reason for this is that its graphing effect does not generate spherical solubility volumes when the plot uses the unit values of $\delta_{L}$ (Figure 1A–C: ellipses dashed in red), except when multiplied by 4 [3]. Hansen proposed that this behaviour is due to the momentary nature of the London atomic forces, unlike the others, which are permanent [3,28].

The value of the interaction radius (Ro) is usually obtained experimentally after plotting the Ra values, of which the highest values for miscible solvents will compose the Ro value of the solute [29,30], as indicated in Figure 1. The value can also be estimated from a solubility database of a range of solvents using computational tools, where a correlation that uses the HSPs of these solvents with that of the solute in question is performed, and the Ro of the solute is proposed [31,32].

From the solubility sphere, compatible solvents are encompassed in the three-dimensional space of the solute, while immiscible solvents are presented externally (Figure 1D,E). Using the relative energy difference (RED), obtained via the ratio between Ra and Ro (Equation (9)), it is possible to estimate whether a determined compound is located in the three-dimensional solubility space of the solute (Figure 1E). If a RED value tending to zero is obtained, it will be a perfect solvent for solvation because of the close HSP values representing a minimum energy difference between species. RED less than 1 indicates high affinity; values equal to or close to 1 suggest a limit condition, which is defined as a border region; and values higher than 1 indicate low chemical affinity, progressively reduced as the value moves away from 1 [2,3,33,34,35].

(9) RED=Ra/Ro

From the RED value, it is possible to predict the compatibility or dispersion between substances and the validity of the classic rule “like dissolves like”. Certainly, there are errors associated with border regions, such as the possible existence of some insoluble solvent inside the Hansen sphere of the solute (RED < 1) as well as a soluble solvent outside the sphere (RED > 1). Deviations are more frequent when involving larger molecular species, since species of smaller molecular volumes have higher solubility [3]. However, deviations can also result from questionable physical data, such as latent heat of vaporisation or dipole moment, obtained outside the reference conditions or from substances that adopt variable conformations dependent on the medium (e.g., temperature, pressure, nature of the solvent, and pH value). In the absence of Ro, Das [35] used solvents miscible with the solute, which mathematically denotes the value of the constant added in Equation 8 and, therefore, similar values between the intermolecular forces of the solvent and the solute, resulting in RED < 1. For the author, Ra > 8 indicates incompatibility (RED >> 1), and those between 4 and 8 indicate border values. In general, the authors are unanimous in saying that the predictions of candidate solvents need to be experimentally confirmed. Therefore, as a tool, HSP does not provide absolute answers but has been used as a predictive indicator for solvent selection as a function of desirability on the solubility of the target solute. If correctly characterised in terms of HSPs ($\delta_{L}$, $\delta_{P}$, and $\delta_{H}$) and Ro, there are high chances of finding the appropriate solvent (RED < 1 and Ra < 4) for the complete solvation of the target analyte, which should then be experimentally validated.

## 3. Manual and Computation Tools to Obtain HSPs

To obtain the solubility parameters ($\delta_{L}$, $\delta_{P}$, and $\delta_{H}$), Hansen used the group contribution methods to correlate the experimental data (molar volume, latent heat of vaporisation, dipole moment, boiling temperature, etc.) of hundreds of molecules (solvents, plasticisers, polymers, pesticides, phytochemicals, and others). Thus, he obtained predictive equations (Equations (1)–(3)) that have coefficients and/or constants for each molecular fragment (*k*), which together result in the total cohesion energy ($E_{coh}$) of the structure in question. Other authors have done the same for the target molecules in their research [27,32,36,37,38]. It is not our objective to review these predictive models; however, to illustrate their use, the method described by Bouteloup and Mathieu [39] and Mathieu [40] was chosen, which allows the user to estimate HSPs manually.

For the dispersion parameter ($\delta_{L}$), Mathieu [40] correlated the reference values of several substances from known HSPs with experimental data associated with their molar volume ($V_{m}$) and molar refractivity ($R_{D}$), obtaining Equation (10) with a determination coefficient (R^2^) of 0.91.

(10) $$\delta_{L}^{2}=93.8+2016{\left(\frac{R_{D}}{V_{m}}\right)}^{2}+\frac{75,044}{V_{m}}{\left(\frac{R_{D}}{V_{m}}\right)}^{2}$$

The molar volume ($V_{m}$) is the data available in the literature for most commercially available compounds, with units cm^3^ mol^−1^ [40], which can be obtained from the sum of the fragment volumes ($V_{k}$) that the molecule has (Equation (11)). Each fragment is dependent on the atomic number of the bonding atom (*Z*), its coordination number ($n_{k}$), and the number of chemically bonded hydrogen atoms ($n_{H}$). For molecules containing saturated/unsaturated or aromatic rings, a volumetric correction constant (Vr) is added (Equation (12)), where n < 5, n = 5, n = 6, and n > 6 represent the number of rings present in molecules with less than 5, equal to 5 or 6, and more than 6 atoms, respectively, and the term $n_{a}$ describes the contribution of aromatic rings [39]. Table 1 presents information about the additive fragments (k), and Table 2 presents the values of the corrections associated with the rings.

(11) $$V_{m}=\sum V_{k}\left(Z,n_{k},n_{H}\right)+Vr$$

(12) $$Vr=n_{<5}V_{<5}+n_{5}V_{5}+n_{>6}V_{>6}+n_{a}V_{a}$$

The molar refractivity ($R_{D}$) of a substance is directly associated with its polarisability, that is, its ease of distorting the electron cloud while interacting with another molecule. By analogy, $R_{D}$ is also the result of the van der Waals atomic radius and is obtained by summing the molar refractivity of each fragment of the molecule ($R_{k}$) (Equation (13)). The additive fragments and their respective molar refractivity increments are presented in Table 3.

(13) $$R_{D}=\sum R_{k}$$

To obtain $\delta_{P}$ and $\delta_{H}$, Mathieu [40] applied Equation (4) in an additive way, that is, the sum of the energies of the atoms or group of atoms ($E_{k}$) that make up the molecule, divided by its molar volume, resulting in the polar ($E_{P}$) and hydrogen ($E_{H}$) cohesive energies (Equation (14)). Table 3 and Table 4 group the values of $E_{k}$ to estimate $E_{P}$ and $E_{H}$, respectively, also obtained using a mathematical correlation between experimental and theoretical values.

(14) $$\delta_{P/H}^{2}=\frac{E_{P/H}}{V_{m}}=\frac{\sum E_{k}}{V_{m}}$$

In Sections S1–S2 of the Supplementary Information, there are examples of the application of Equations (10)–(14) and Tables S1–S4 to obtain the HSPs for two compounds, where it was proven that the bases for the calculations were reliable and led to the correct prediction of the HSPs for the evaluated analyte. Although it is possible to perform these calculations manually, any error or distortion in the value is perpetuated. Therefore, a search of the literature is convenient for verifying whether they are available [3,4,29,30].

Once the HSPs for the target solute have been obtained, the search for the most suitable solvent must be performed, which can be tedious, time-consuming, and error prone. To avoid these issues and optimise the work, Excel spreadsheets and software have been developed to provide $V_{m}$, the HSPs, and Ro of the target analyte and a list of solvents, non-solvents, and solvent mixtures with RED and other information relevant to the analyst’s decision-making process. These tools support the extraction, separation, or isolation stage of target phytochemical analyte(s), among countless other applications.

**Figure 2:**
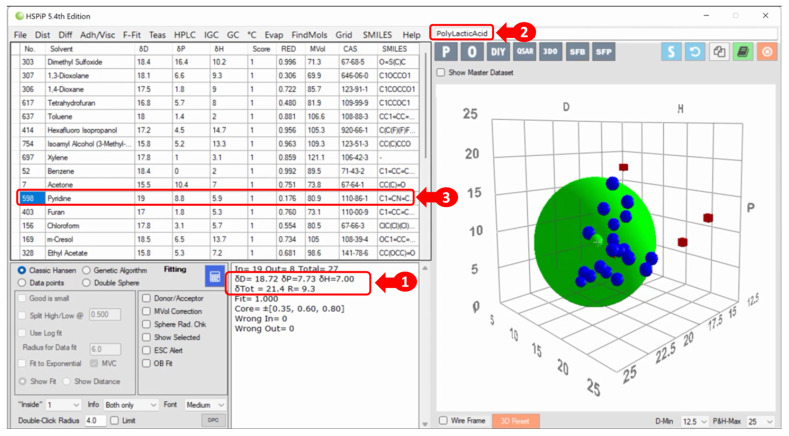
HSPiP in its 5.4 edition presents HSP information (1) about the target analyte: lactic acid polymer (2). In the 3D graph, the analyte’s green solubility sphere with the interaction radius is superimposed on the blue spheres (compatible solvents, RED < 1), and the red squares are located outside the sphere (incompatible solvents). A list of solvents and their RED values are provided with a highlight (3) for pyridine due to its lower RED value [2].

In 2008, Hansen Solubility Parameters in Practice (HSPiP) was introduced to the market. A triple product (software, e-Book, and datasets) that provides the semi-empirical predictive power of Hansen’s solvation and thermodynamic properties for over 10,000 substances is available, adding simplicity to the practical work for a suitable selection of the extraction solvent [33,41]. For the target analyte, the software lists and orders candidate solvents by their chemical affinity (lowest RED) and illustrates the solvation power by HSP spheres in a 3D graph (Figure 2). If the target molecule is not listed in the HSPiP, it is possible to draw the molecular structure or enter its Chemical Abstracts Service ID (CAS), Simplified Molecular Input Line Entry Simplification (SMILES), or MolFile notation, with which the HSPs can be estimated based on the sequence of fragments (k) that the substance presents [2,4,34,41,42,43]. Furthermore, it is possible to optimise solvent mixtures (up to eight solvents) for an efficient choice in place of the previously selected pure solvent [41]. For this, Equation (15) is employed, where $x_{n}$ is the volumetric fraction of the n solvents present in the mixture and $\delta_{X}$ one of the three HSPs, which must be calculated for all of them.

(15) $${\Delta x}_{Mix}=x_{1}\delta_{1}+x_{2}\delta_{2}+x_{3}\delta_{3}+\cdots +x_{n}\delta_{n}$$

Other predictive tools have been developed and used as alternatives to HSPs, including Abraham solvation parameters [44], the conductor-like screening model for real solvents (COSMO-RS), linear solvation energy relationships introduced by Kamlet, Abboud, and Taft (KAT-LSER) [45], the extended Hildebrand solubility approach (EHSA) [46,47], and the Jouyban–Acree model [48,49]. Some are being used in combination to increase accuracy in the most suitable solvent for the extraction of phytochemicals and other molecules. However, a discussion of their fundamentals and applications is not within the scope of this work.

## 4. HSPs Applied to Phytochemical Extraction Methods

Since the advent of HSPs, there has been growing interest in the method by the academic and industrial sectors, enabling the development of more selective, efficient, sustainable, and safe phytochemical prospecting procedures. In this section, concrete examples of the use of HSPs in phytochemical extraction methods are discussed.

### 4.1. Solid Phase Extraction

#### 4.1.1. Soxhlet Extraction

Soxhlet extraction is usually employed for the extraction of total lipids or lipid components from vegetable or animal matrices using *n*-hexane as the extracting solvent [50,51]. Although *n*-hexane is a volatile solvent (boiling point (BP) is 69 °C), low cost (USD 65 L^−1^), and easily obtained due to its petrochemical origin, it is toxic, non-renewable, and has a high environmental impact [52]. Therefore, HSPs have been used to find a more ecological and sustainable alternative to replacing *n*-hexane. In this sense, Bertouche et al. [53] used HSPs to select a biologically based solvent for the extraction of fatty acids of varying sizes and degrees of unsaturation (C_16:0_, C_17:0_, C_18:0_, C_20:0_, C_22:0_, C_16:1_, C_18:1_, C_20:1_, C_18:2_, and C_18:3_) from peanut, soybean, sunflower, and olive seeds. The authors chose to work with α-pinene, a monoterpene of natural origin, a constituent of the fixed and essential oils of coniferous trees, with antibiotic, antimetastatic, antimicrobial, and apoptotic activity [54,55]. Lower RED values supported the choice for α-pinene, which was validated by the higher extraction yields (24.5% for olive, 42.3% peanuts, 21.1% soya, and 67.2% sunflower), about 7.1% to 27.8% higher than extraction with *n*-hexane (22.6% for olive, 39.5% peanuts, 19.5% soya, and 52.6% sunflower), indicated by the greater polarity and diffusivity of α-pinene, probably aided by the higher boiling temperature of this biosolvent (156 °C–158 °C). Although greater energy expenditure is required to boil α-pinene in Soxhlet extraction, there is less loss using evaporation and, therefore, greater solvent recovery was observed, about 90% against 50% for *n*-hexane. This recovery means that for every 10 extractions, 5 L of *n*-hexane will be consumed to replace the solvent with 1 L of α-pinene. The authors performed GC-MS analysis and found that no degradation products were detected and that the change in fatty acid composition was insignificant. However, other information should be considered, such as the cost of this biosolvent (USD 555 L^−1^), which may make its practical application unfeasible, even considering the difference in the recovery of both solvents, not to mention the energy expenditure.

Other authors have also reported the use of HSPs and RED to find a substitute for *n*-hexane in the extraction of lipids via Soxhlet. Li et al. evaluated the performance of several solvents (butanol, ethanol, isopropanol, α-pinene, p-cymene, and D-limonene) for canola oil extraction [56]. The authors found p-cymene to have a higher extraction yield, 52.8% more oil than *n*-hexane, followed by D-limonene (50.8%), isopropanol (42.8%), butanol (34.6%), and α-pinene. (12.6%), while ethanol extracted less oil than *n*-hexane. In the evaluation, p-cymene extracted more polar lipids (free fatty acids (FFA), monoglyceride (MAG), and diglyceride (DAG)) than *n*-hexane, although it extracted fewer tocopherols and an equivalent number of sterols. With the same purpose and matrix, Sicaire et al. theoretically and experimentally evaluated the use of 2-methyltetrahydrofuran (2-MeTHF), a solvent obtained from biomass, which resulted in an oil yield and composition of fatty acids and tocopherols equivalent to *n*-hexane but with less extracting power for sterols [57]. The authors verified that the energy of 2-MeTHF is viable for its use on an industrial scale since it has physical properties with slightly higher values (e.g., BP 80 °C), in addition to having a toxicity index of 4, lower than *n*-hexane (5) although it is 3.9 times more expensive.

#### 4.1.2. Solid–Liquid Extraction (SLE) via Maceration

Yara-Varón et al. [58] employed HSPs to find a green substitute to replace *n*-hexane in the extraction of carotenes (mainly composed of α-carotene and β-carotene, 1:2) in carrots (*Daucus carota*). Based on a predictive computer program, 18 solvents were suggested: 5 terpenes (α-pinene, β-pinene, β-cymene, β-myrcene, and D-limonene), 5 esters (methyl acetate, ethyl acetate, ethyl laurate, ethyl oleate, dimethyl carbonate (DMC), and isopropyl palmitate), cyclopentyl methyl ether (CPME), 2-methyltetrahydrofuran (2-MeTHF), 4 alcohols (isopropyl alcohol, 1-butanol, ethanol, and methanol), and water. Not all solvents were experimentally evaluated since the boiling point, the energy required for evaporation, and toxicity, in addition to solubility, were considered in the choice. Although terpenes are the compounds with the lowest RED value, their high boiling points (157.8–385.9 °C) eliminate them, as well as ethyl laurate esters (269.0 °C) and isopropyl palmitate (340.7 °C). In contrast, low boiling points eliminated methyl acetate (57.1 °C), which could result in losses during the extraction process and difficult recovery. More polar solvents, such as water, methanol, ethanol, and 1-butanol, were also excluded due to solubility (RED value > 3.8), resulting in five candidates: 2-MeTHF, CPME, DMC, ethyl acetate, and isopropyl alcohol. For the chosen solvents, SLE via maceration was used, a classic method for extracting phytochemicals. This was performed on 30 g of dry and ground sample added to 125 mL of solvent in a reactor heated to 65 °C under constant agitation and maceration for 1 h. The extract was collected after filtration and added to another two volumes of 125 mL used to wash the solid residue. Yields are presented in Table 5, as well as other deciding factors.

Table 5 shows an excellent correlation between the RED values for the chosen biosolvents and the obtained extraction yields, although *n*-hexane should extract with a yield similar to that of 2-MeTHF. The authors also used COSMO-RS as an alternative predictive tool to HSPs. However, they could not predictively rank the selected solvents, equivalently suggesting them. Lastly, the authors pointed out CPME, 2-MeTHF, and ethyl acetate as promising green solvents for replacing *n*-hexane, due to the extraction yield, energy cost for evaporation, toxicity, and source (Table 5).

#### 4.1.3. Ultrasound-Assisted Extraction (UAE)

UAE is an interesting method for phytochemical extractions. For successful extraction, it is necessary to find the ideal solvent and, preferably, a greener solvent, making the process ecologically correct. Shekaari et al. [9] applied HSPs together with UAE to obtain curcuminoids, polyphenolic phytochemical compounds present in *Curcuma longa* Linn. For this, several deep eutectic solvents (DESs) and ionic liquids (ILs) were evaluated and compared with conventional solvents, correlating HSPs with extraction yields. The authors found higher extraction yields when using these non-conventional solvents (5–16% *m*/*m*) compared to commonly used solvents (2–5% *m*/*m*) (Table 6). The reason for this behaviour is directly related to the respective HSPs and Ra values for the evaluated solvents. That is, the lower the Ra value, the greater the yield obtained. Therefore, HSPs-Ra indicates a strong interaction between curcuminoids and DES choline chloride/malonic acid (1:1) compared to other solvents, thus suggesting the best solvent for extraction (Ra = 3.97 and a yield of 16.45% *m*/*m*). Hu et al. [59] showed that ChCl/MA can be recovered using evaporation at 80 °C under a vacuum, and once recycled, it can be reused four more times without losing efficiency. With HSPs, the effect of ILs is observed due to the hydrophobic chain and hydrophilic head group, as the solubility of curcuminoids with DESs is better and higher than that of imidazole ILs, which shows that the π-π interaction is not an imperative factor. Research shows that the electrical charge of the main group of solvents plays an important role in electrostatic interactions with curcuminoids. These results are further supported by the HSP calculation and are consistent with the experimental results of solubility and extraction, in which the inverse relationship between Ra and extraction yield was confirmed, this time for ILs and DESs (Table 6).

#### 4.1.4. Pressurised Liquid (or Accelerated Solvent) Extraction (PLE)

PLE has been employed together with the HSPs approach to target phytochemical recovery from several natural sources [60,61,62]. High pressure leads to high extraction efficiency, with less solvent consumption in a shorter time, allowing the use of greener solvents [63,64,65,66]. In this context, Ballesteros-Vivas et al. [67] applied sequential PLE for (*i*) lipids and (*ii*) polyphenol recovery in mango (*Mangifera indica* L.) seeds. Heptane was experimentally selected as a solvent for the first step (non-polar extract) of the PLE procedure, while the second extract used the HSPs approach for solvent selection and response surface methodology and other variables to maximise the mangiferin content. Mangiferin HSP data and green solvents were obtained using HSPiP, indicating ethyl lactate as the most miscible after ethanol, ethyl acetate, and (+)-limonene. However, ethyl lactate has a higher boiling point (154 °C), which burdens its evaporation. Therefore, the authors used Equation 14 to find an ethanol/ethyl acetate 50:50 *v*/*v* mixture close to ethyl lactate’s RED. These data were consistent with the experimental results of the highest mangiferin content.

#### 4.1.5. Effect of Extraction Methodologies

To select a solvent and the most suitable technology for the extraction of cynaropicrin, a guaianolide sesquiterpene lactone from *Cynara cardunculus* var. *altilis* (cardoon) leaves, Brás et al. [43] resorted to HSPs and SLE, MAE, PLE, and UAE as extraction methods compared to Soxhlet extraction. The solvents chosen for the extraction were selected from the RED values and experimentally evaluated, keeping a constant liquid/solid ratio (16 mL g^−1^) and temperature (40 °C), except for MAE because of the intrinsic solvent heating. Table 7 summarises the results.

The experimental SLE results indicated good agreement with HSP-RED values, except for ethanol, of which the analytical error discredited the measure. The authors did not explain the RED yield deviation but suggested an effect caused by the complex plant matrix in the prediction. In contrast, MAE and PLE extractions agree with HSP predictions, while the UAE resembles the behaviour of SLE. In general, 100% ethanol and ethyl acetate resulted in the highest extraction yields for all extraction techniques, except for MAE, due to analyte degradation caused by the overheating of the system (analyte-solvent). The authors decided to use ethanol—the less toxic solvent evaluated—in the extraction via ultrasound, a decision supported by its lower energy consumption and shorter extraction time.

### 4.2. Liquid–Liquid Extraction (LLE)

To establish an isolation method for cafestol and kahweol (C&K), two dialcohol-phytochemicals from Arabica coffee beans with elevated commercial value (standards quoted as USD 3400 g^−1^ and USD 10,800 g^−1^, respectively), Novaes et al. optimised the saponification reaction of these natural esterified compounds and used HSPs to choose the better solvent for the extraction of diterpene alcohol mixture (C&K) [68]. The authors applied the HSPiP software to predict the miscibility of C&K in a list of solvents to LLE with high recovery in the gram scale. The solvent could not be miscible in water, added to stop the saponification reaction composed of methanolic KOH solution, and should have a low boiling point to facilitate its evaporation. Dichloromethane (DCM), ethyl acetate, and *tert*-butyl methyl ether (TBME) matched the requirements and were tested. Ethyl acetate was soluble in the alkaline solution. DCM had lower Ra values and acceptable extraction efficiency (1.27 g of C&K 100 g^−1^ of green coffee beans). TBME had better recovery extraction of both diterpenes (1.45 g). The authors selected methanol as the HPLC mobile phase to isolate each diterpene for purification according to HSPs, solvent price, and the ease of vacuum removal at room temperature. Notably, 100% methanol was not able to separate the analytes in a single analysis; therefore, five cycles in a preparative liquid chromatography system were successfully used to obtain these phytochemicals with analytical purity.

The whole procedure was scaled up by increasing the coffee bean mass (from 0.667 to 300 g) and all reagents and solvents (from 2 to 800 mL) to obtain the diterpenes in g-scale, resulting in 1.55 g per extraction. The increase in scale represented a reduction in the extraction efficiency, which was to be expected due to the greater amount of materials and the difference in the efficiency of the equipment (e.g., form and speed of stirring, mass and heat transfer, and equipment dimensions). Therefore, as is usual in scale-up, the procedure needs to be further optimised.

### 4.3. Other Extraction Techniques

For other extraction techniques employed with HSP solvent selection in the extraction of phytochemicals, see Table S5 in the Supplementary Information.

## 5. Beyond the Limits of HSPs

**Figure 3:**
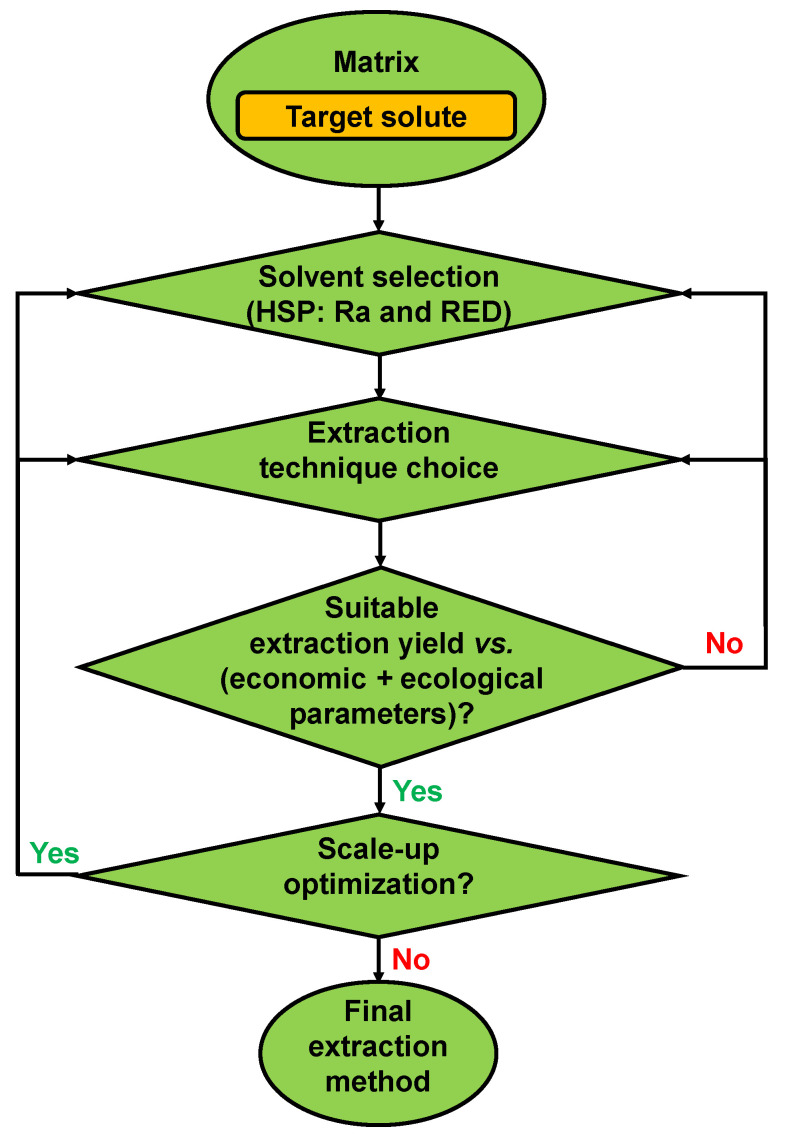
Flowchart to refine phytochemical extraction.

Beyond the best extraction properties, many other criteria are involved in the appropriate solvent selection. For example, Figure 2 shows pyridine (RED = 0.176) as an excellent solvent for the analyte in question, but it has an unpleasant odour; 1,3-dioxolane (RED = 0.306) would also be a good solvent, but it is very volatile; and dichloromethane would be acceptable (RED = 0.373), but it is chlorinated. In these situations, the analyst needs to evaluate other characteristics of the solvent before selection, which will also depend on the matrix studied. Therefore, for the extraction step, the analyst must consider the chemical selectivity of the solvent to avoid additional steps in the purification of the analyte [8,41]. The solvent boiling point should be low enough for easy elimination, having low energy consumption but not too volatile to avoid loss and extractive deficiency. A good choice will facilitate the recovery of the analyte and solvent for use on an industrial scale [68]. In addition to the best extraction properties, it is essential to consider environmental and economic aspects when choosing a solvent. Ecologically friendly alternatives should be sought. It is also ideal to have solvents with low or no toxicity, flammability (high flash point), and corrosive power and, if possible, biobased and food grade, with a low global warming potential index and affordable cost [8,9,41,66,69]. Economic viability is also crucial in seeking accessible and cost-effective solutions, considering energy consumption and other resources involved in the process [70]. Other properties, such as density, viscosity, chemical stability, shelf life, availability, and the possibility of reuse and recovery, as well as the method, logistics, and costs for disposal, must be evaluated before the final section is made, such as the extraction technique to be used [41,43,66,68,69]. Figure 3 is a flowchart of the process of refining phytochemical extraction before the analyst makes the final decision.

## 6. Conclusions

HSPs have proven to be a theoretical tool for predicting solubility and selecting solvents for target phytochemical extraction. This review presented HSPs from the perspective of the author’s history, intermolecular forces (induced dipole, permanent dipole, and hydrogen bonding), equations, and graphical modelling. The methods of contribution by groups are used to determine HSPs, allowing for manual and computational estimations that support accessible and faster prediction, respectively.

This review also presented real examples of phytochemical extraction procedures that combine HSPs: solid–liquid extraction via maceration, Soxhlet, microwave-assisted extraction, and pressurised liquid extraction, among others. HSPs have made it possible to indicate possible solvents but not the better choice, which must consider the solvent security and health aspects, such as its physical–chemical properties (boiling point, chemical stability, corrosive power, density, flammability, and viscosity), type of bio or chemical source, and economic parameters (availability, shelf life, recycling, and cost), all of which are associated with the suitable extraction yield based on extraction technique choice and its optimisation step. 

## Figures and Tables

**Table 1:** Additive fragments (k), coordination number ($n_{k}$), number of bonded hydrogen atoms ($n_{Hk}$), volume ($V_{k}$), and standard deviation ($\sigma_{k}$). Adapted from Bouteloup and Mathieu [39].

k	$n_{k}$	$n_{Hk}$	$V_{k}$	$\sigma_{k}$
−Br	1	0	27.52	0.15
≡C	1	0	14.68	1.57
−C≡	2	0	9.89	0.23
≡CH	2	1	22.77	0.46
>C=	3	0	0.00	0.12
=CH-	3	1	13.23	0.06
=CH_2_	3	2	27.17	0.17
>C<	4	0	−8.40	0.20
-CH<	4	1	4.46	0.13
>CH_2_	4	2	16.57	0.02
-CH_3_	4	3	29.58	0.08
-Cl	1	0	24.74	0.09
≡N	1	0	14.09	0.35
-N=	2	0	7.42	0.18
=NH	2	1	18.14	0.44
-N<	3	0	−3.08	0.19
>NH	3	1	7.74	0.22
-NH_2_	3	2	17.81	1.18
=O	1	0	14.89	0.12
>O	2	0	6.25	0.07
-OH	2	1	11.78	0.13
-P<	3	0	10.42	0.60
>P<	4	0	−1.94	0.44
−PH<	4	1	10.06	1.57
=S	1	0	25.92	0.50
>S	2	0	14.90	0.22
−SH	2	1	26.14	0.30
>S=	3	0	5.58	1.75
>S<	4	0	−3.74	0.48

**Table 2:** Fixes associated with rings. Adapted from Bouteloup and Mathieu [39].

Increment	Ring Correction, V	Standard Deviation, σ
*V < 5*	10.89	0.32
*V5*	9.41	0.23
*V6*	6.89	0.22
*V > 6*	3.75	0.56
*Va*	1.82	0.36

**Table 3:** Parameters required to estimate $\delta_{P}$ via Equation (14). Adapted from Mathieu [40].

Fragment (k)	J/mol	Fragment (k)	J/mol
>NH	2783	>C=O	7492
-NH_2_	8235	-COOH	−5494
Nitro	13,276	Carbonate	19,019
-O-	1603	>C=O (amide)	15,972
-OH	4125	>C=O (ester)	3653
-Cl	1637	-CN	16,053
P=O	20,310		

**Table 4:** Parameters required to estimate $\delta_{H}$ via Equation (14). Adapted from Mathieu [40].

Fragment (k)	J/mol	Fragment (k)	J/mol
>CH-	24.5	-O-	1980
=N-	3252	-OH	16,945
>NH	−1576	-OH (COOH)	7094
>NH (amide)	5060	X (F, Cl, Br, I)	412
-NH_2_	5484		

**Table 5:** HSP-RED selected solvents and their technical properties, total carotene (Car) extraction yield, and ecological parameters after Yara-Varón et al. [58].

Solvent	RED		Boiling Point (°C)	Energy Evaporation (kWh kg^−1^ Solvent)	Toxicity Index	Resource	Total Yield (mg 100 g^−1^)
	α-Car	β-Car					
*n*-Hexane	1.31	1.34	68.8	0.121	5	Petroleum	55.8 ± 7.6
CPME	1.14	1.33	105.3	0.132	4	Chemical synthesis	78.4 ± 7.4
2-MeTHF	1.51	1.33	79.9	0.126	4	Cereal crop	65.8 ± 4.8
Ethy acetate	2.10	1.95	73.9	0.127	5	Cereal crop	53.1 ± 8.2
DMC	2.69	2.51	90.5	0.194	5	Chemical synthesis	37.8 ± 4.6
IPA	3.69	3.56	73.0	0.219	5	Cereal crop	40.8 ± 5.5

**Table 6:** The extracted amounts of curcuminoids using general solvents (GS), three ionic liquids (ILs), and deep eutectic solvents (DESs), and their HPS values and common properties. Adapted from Shekaari et al. [9].

Name (Molar Ratio)		Molar Mass (g mol^−1^)	$\delta_{L}$	$\delta_{P}$	$\delta_{H}$	$\delta_{Total}$	Ra	Yield (*%*)
Analyte	Curcuminoids	368.40	17.86	4.01	11.86	21.81	-	-
GS	Acetone	58.08	15.50	10.40	7.00	19.90	9.31	3.28
GS	Ethanol	46.08	15.80	8.80	19.40	26.50	9.84	4.85
GS	*n*-Hexane	86.18	14.90	0.00	0.00	14.90	13.85	5.65
IL	1-Butyl-3-methyl imidazolium chloride	174.67	20.12	10.63	9.38	24.61	8.39	5.94
IL	1-Hexyl-3-methyl imidazolium chloride	202.72	19.40	8.36	8.31	24.61	6.40	7.75
IL	1-Octyl-3-methyl imidazolium chloride	230.78	18.94	6.88	7.54	21.52	5.62	10.63
DES	Choline chloride/Glycerol (1:2)	107.93	16.54	5.45	21.98	28.04	10.55	4.60
DES	Choline chloride/Ethylene glycol (1:2)	87.92	15.88	5.34	19.50	25.71	8.71	5.09
DES	Choline chloride/Ascorbic acid (2:1)	151.88	16.43	4.83	18.69	25.35	7.45	6.03
DES	Choline chloride/Citric acid (1:1)	165.87	16.31	5.18	17.59	23.58	6.62	7.50
DES	Choline chloride/Glycolic acid (1:2)	97.24	19.72	6.01	17.96	27.34	7.41	7.64
DES	Choline chloride/Lactic acid (1:2)	106.60	16.07	4.63	16.63	23.58	6.00	8.02
DES	Choline chloride/Acetic acid (1:2)	86.57	15.76	4.95	13.44	21.30	4.58	10.74
DES	Choline chloride/Oxalic acid (1:1)	114.83	16.26	5.71	14.44	22.48	4.45	11.22
DES	Choline chloride/Propionic acid (1:2)	95.93	15.88	4.39	12.57	20.77	4.04	12.09
DES	Choline chloride/Malonic acid (1:1)	114.83	16.30	5.32	13.94	22.09	3.97	16.45

**Table 7:** HSP-RED-selected solvents and their cynaropicrin extraction yield (mg g^−1^ of dry weight) for different extraction methods, time (min), and energy consumption (kWh g^−1^ of cynaropicrin). Adapted from Brás et al. [43].

Solvent	RED	SLE 60 min 1.16 kWh g^−1^	MAE 15 min 0.74 kWh g^−1^	PLE 5 min 0.18 kWh g^−1^	UAE 5 min 0.03 kWh g^−1^
Ethanol	1.44	56.9 ± 81.5	30.8 ± 1.2	47.9 ± 3.6	55.0 ± 2.9
Ethanol/water (4:6)	2.90	18.7 ± 2.6	18.5 ± 1.5	39.6 ± 3.5	41.4 ± 3.0
Ethyl acetate	0.36	37.5 ± 2.2	38.4 ± 1.6	57.0 ± 4.5	52.6 ± 1.7
Water	3.89	13.6 ± 1.1	1.47 ± 0.2	-	23.8 ± 1.6
Dichloromethane *	0.49	40.3 ± 1.1 mg g^−1^ in 7 h of Soxhlet extraction			

SLE: solid–liquid extraction; MAE: microwave-assisted extraction; PLE: pressurised liquid extraction; UAE: ultrasound-assisted extraction; * Dichloromethane as reference.

## Supplementary Materials

The following supporting information can be downloaded at: https://www.mdpi.com/article/10.3390/plants12163008/s1: Section S1: Paracetamol (CAS No: 103-30-2). Table S1: Parameters required to estimate paracetamol $V_{M}$ and $R_{D}$ via Equations (10)–(13); Table S2: Parameters required to estimate $E_{P}$ and $E_{H}$ of paracetamol via data from Table 3 and Table 4 on Equation (14). Section S2: Salicylic acid (CAS No: 69-72-7). Table S3: Parameters required to estimate salicylic acid ($V_{M}$ and Ro) via Equations (10)–(13); Table S4: Parameters required to estimate $E_{P}$ and $E_{H}$ of salicylic acid via data from Table 3 and Table 4 on Equation (14). Section S3. Other extraction techniques. Table S5: Sampler of extraction techniques employed with HSP solvent selection for the extraction of phytochemicals [71,72,73,74,75,76].
