# Supplementary material for: Hansen Solubility Parameters Applied to the Extraction of Phytochemicals
Source: Plants (Basel). 2023 Aug 21;12(16):3008. doi: 10.3390/plants12163008 (PMC10459436; doi:10.3390/plants12163008)
Supplement: Supplementary file 1 [file plants-12-03008-s001.zip › plants-2510667-supplementary.pdf]

Supporting Information – Plants

HANSEN SOLUBILITY PARAMETERS APPLIED TO THE EXTRACTION OF PHYTOCHEMICALS

Fábio Junior Moreira Novaes[1], *Daliane Cláudia de Faria*[1], *Fabio Zamboni Ferraz* [1], *Francisco Radler de Aquino Neto*[2,*]

[1] Universidade Federal de Viçosa, Departamento de Química, Avenida Peter Henry Rolfs, s/n, Campus Universitário, Viçosa, MG 36570-900, Brazil

[2] Universidade Federal do Rio de Janeiro, Instituto de Química, Laboratório de Apoio ao Desenvolvimento Tecnológico (LADETEC), Avenida Horácio Macedo, 1281, Polo de Química, bloco C, Rio de Janeiro, RJ 21941-598, Brazil

Content:

S1. Example of obtaining HSPs for the paracetamol molecule

S2. Example of obtaining HSPs for the salicylic acid molecule

S3. Other extraction techniques

S1. Paracetamol (CAS No: 103-30-2)

In order to exemplify the use of Equations 10-13 and Tables 1-2 of the main text, **Table S1** presents the calculations for obtaining the volume (V_m) and molar refractivity (R_D) of paracetamol.

Table S1. Parameters required to estimate paracetamol V_m and R_D via Equations 10-13.

Chemical structure	*Fragment* (k)	n	V_k	$\sum V_k$	R_k	$\sum R_k$
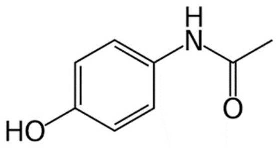	-OH	1	11.78	11.78	2.51	2.51
	=CH- [a]	4	13.23	52.92	4.46	17.84
	>C= [a]	2	0.00	0.00	3.48	3,69
	V_6	1	6.89	6.89	-	-
	V_a	1	1.82	1.82	-	-
	>NH	1	7.74	7.74	3.69	3.69
	>C=	1	0.00	0.00	3.15	3.15
	=O	1	14.89	14.89	1.84	1.84
	$-CH_3$	1	29.58	29.58	5.74	5.74
				V_m = **125.62**		R_D = **41.73**

[a] referring to the aromatic ring; n: number of occurrences; V_k fragment volume; R_k fragment refractivity.

The calculated molar volume (125.62) is close to the experimental value of 120.9 ± 3.0 cm^3/mol, as the molar refractivity. (41.27 *vs.* 42.4 ± 0.3) [1], indicating that the approach put forward by Bouteloup & Mathieu [2] and Mathieu [3] are acceptable to estimate the V_m and R_D, respectively, and consequently to obtain the Hansen dispersion parameter (δ_D) by using Equation 10:

$$\delta_D = \sqrt{93.8 + 2016 \times \left(\frac{41.73}{125.62}\right)^2 + \frac{75044}{125.62} \times \left(\frac{41.73}{125.62}\right)^2} = 19.5$$

To calculate the sum of polar (E_P) and hydrogen (E_H) cohesive energies, data from Tables 3 and 4 of the main text are used, respectively, based on Equation 14. The determination of such energies is compiled in **Table S2**.

Table S2. Parameters required to estimate E_P and E_H of paracetamol via data from Tables 3 and 4 on Equation 14.

**Chemical structure**	***Fragment* (k)**	$\boldsymbol{n}$	$\boldsymbol{E_{P_k}}$	$\boldsymbol{\sum E_{P_k}}$	$\boldsymbol{E_{H_k}}$	$\boldsymbol{\sum E_{H_k}}$
	-OH	1	4125	4125	16945	16945
	=CH-[a]	4	-	-	24.5	98
	>C= [a]	2	-	-	-	-
	>NH	1	2783	2783	5060	5060
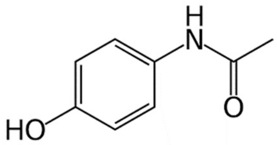	*N*	1	-	-	3252	3252
	>C=	1	15972	15972	-	-
	=O	1	1603	1603		
	O	2	-	-	1980	3960
	>CH-	3	-	-	24.5	73.5
				$\boldsymbol{E_P}$ = **24483**		$\boldsymbol{E_H}$ = **29388.5**

[a] referring to the aromatic ring; n: number of occurrences; E_{P_k} polar energy of fragment (*k)*; E_{P_k} hydrogen bonding energy for the *k* fragment.

From the respective cohesion energies, the polar and hydrogen bonding HSPs are obtained using Equation 14:

$$\delta_P = \sqrt{\frac{E_P}{V_m}} = \sqrt{\frac{24483}{125.62}} = 13.96$$

$$\delta_H = \sqrt{\frac{E_H}{V_m}} = \sqrt{\frac{29388.5}{125.62}} = 15.3$$

The HSP values estimated above ($V_m = 125.62,\ \delta_D = 19.5,\ \delta_P = 13.9\ e\ \delta_H = 15.3$) are not all equivalent to those described by Hansen (2007, p. 451: $V_m = 151.2,\ \delta_D = 17.8,\ \delta_P = 10.5, \text{and}\ \delta_H = 13.9$) [4], which has a discrepant molar volume about the experimental value (120.9 cm^3/mol). The cohesion energies for the Hansen data can be recalculated and the values of each parameter obtained using the experimental volume, whose new values

($\delta_D = 19.9,\ \delta_P = 11.7, and\ \delta_H = 15.5$) do approach those obtained using the methodology of Mathieu.

S2. Salicylic acid (CAS No: 69-72-7)

An equivalent approach performed in item S1 is presented below for the determination of the volume (V_m) and molar refractivity (R_D) of salicylic acid (**Table S3**) and its respective HSPs (**Tabela S4**).

Table S3. Parameters required to estimate salicylic acid (V_m and R_D) via Equations 10-13.

Chemical structure	*Fragment* (k)	n	V_k	$\sum V_k$	R_k	$\sum R_k$
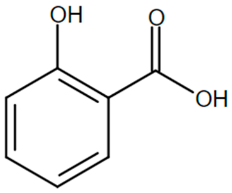	-OH	2	11.78	23.56	2.51	5.02
	=CH-[a]	4	13.23	52.92	4.46	17.84
	>C= [a]	2	0.00	0.00	3.48	6,69
	V_6	1	6.89	6.89	-	-
	V_a	1	1.82	1.82	-	-
	>C=	1	0.00	0.00	3.15	3.15
	=O	1	14.89	14.89	1.84	1.84
				$V_m =$ **100.08**		$R_D =$ **34.54**

[a] referring to the aromatic ring; n: number of occurrences; V_k fragment volume; R_k fragment refractivity.

Once again, the calculated molar volume (100.08 cm^3/mol) is close to the experimental value (100.4 ± 3.0 cm^3/mol), as well as the molar refractivity (34.54 *vs.* 35.1 ± 0.3) [5], confirming the assertiveness of the Bouteloup & Mathieu [2].

$$\delta_D = \sqrt{93.8 + 2016 \times \left(\frac{34.54}{100.08}\right)^2 + \frac{75044}{100.08} \times \left(\frac{34.54}{100.08}\right)^2} = 20.6$$

Table S4. Parameters required to estimate E_P and E_H of salicylic acid via data from Tables 3 and 4 on Equation 14.

**Chemical structure**	***Fragment (k)***	$\boldsymbol{n}$	$\boldsymbol{E_P}$	$\boldsymbol{\sum E_P}$	$\boldsymbol{E_H}$	$\boldsymbol{\sum E_H}$
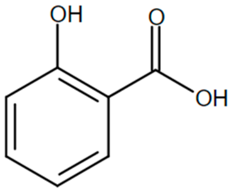	-OH	2	4125	8250	16945	33890
	=CH-[a]	4	-	-	24.5	98
	>C=[a]	2	-	-	-	-
	>C=	1	15972	15972	-	-
	=O	1	1603	1603		
	O	3	-	-	1980	5940
				$\boldsymbol{E_P}$ **= 25825**		$\boldsymbol{E_H}$ **= 39928**

[a] referring to the aromatic ring; n: number of occurrences; E_{P_k} polar energy of fragment (*k*); E_{P_k} hydrogen bonding energy for the *k* fragment.

$$\delta_P = \sqrt{\frac{E_P}{V_m}} = \sqrt{\frac{25825}{100.08}} = 16.06$$

$$\delta_H = \sqrt{\frac{E_H}{V_m}} = \sqrt{\frac{39928}{100.08}} = 20.0$$

Again, the estimated HSP values for salicylic acid ($V_m = 100.08,\ \delta_D = 20.6,\ \delta_P = 16.06, and\ \delta_H = 20.0$) are not all equivalent to those described by Hansen (2007, p. 463: $V_m = 95.7,\ \delta_D = 19.4,\ \delta_P = 10.1\ e\ \delta_H = 17.4$) [4]. However, if recalculated with $V_m = 100.1$, one has $\delta_D = 19.0,\ \delta_P = 9.9$, and $\delta_H = 17.0$, which are acceptable.

S.3. Other extraction techniques

Other extraction techniques have been employed after HSP solvent selection in the extraction of phytochemicals (Table S5).

Table S5. Sampler of extraction techniques employed after HSP solvent selection for the extraction of phytochemicals.

Analite	Matrix	Selected solvent	HSP	Extraction technique	Authors
TAG	*Litsea cubeba* kernel oils	CPME	0.43-0.44 (RED)	Soxhlet	[6]
β and γ-Tocopherols			0.76 (RED)		
δ-Tocopherol			0.63 (RED)		
β-Sitosterol			0.61 (RED)		
Stigmasterol			0.7 (RED)		
Campesterol			0.61 (RED)		
Bilberry oil	Solid waste from bilberry seeds	Norflurane	13.81 (δ_{Total})		[7]
α-Mangostin	*Garcinia mangostana L*	Ethyl acetate	2.31 (RED)	Reflux	[8]
		DMC	2.63 (RED)		
		2-MeTHF	2.19 (RED)		
α-Thujene	Blackcurrant buds	MeTHF	0.65 (RED)	Supercritical fluid	[9]
β-Pinene			0.87 (RED)		
α-Pinene			0.9 (RED)		
β-Myrcene			0.78 (RED)		
α-Phellandrene			0.68 (RED)		
γ-Terpinene			0.47 (RED)		
Terpinolene			0.63 (RED)		
Mangiferin	Mango seed kernel oil	Ethanol:ethyl acetate 50:50 v/v	9.7 (Ra)	Pressurized-liquid	[10]

References

1. ChemSpider: Paracetamol. Available online: http://www.chemspider.com/Chemical-Structure.1906.html (accessed on 02/02/2023).
2. Bouteloup, R.; Mathieu, D. Improved model for the refractive index: application to potential components of ambient aerosol. *Phys. Chem. Chem. Phys.*, **2018**, *20*, 22017−22026.
3. Mathieu, D. Pencil and Paper Estimation of Hansen Solubility Parameters. *ASC Omega*, **2018**, *3*, 17049-17056.
4. Hansen, C.M. Hansen Solubility Parameters: A User's Handbook, 2nd ed.; CRC Press: Taylor & Francis Group: Boca Raton, USA, 2007.
5. ChemSpider: Salicylic acid. Available online: http://www.chemspider.com/Chemical-Structure.331.html (accessed on 02/02/2023).
6. Zhuang, X.; Zhang, Z.; Wang, Y.; Li, Y. The effect of alternative solvents to n-hexane on the green extraction of Litsea cubeba kernel oils as new oil sources. *Ind. Crops Prod.*, **2018**, *126*, 340-346.
7. Cante, R. C.; Prisco, I.; Garella, I.; Gallo, M.; Nigro, R. Extracting the lipid fraction from waste bilberry seeds with a hydrofluorocarbon solvent. *Chem. Eng. Res. Des.*, **2020**, *157*, 174-181.
8. Bundeesomchok, K.; Filly, A.; Rakotomanomana, N.; Panichayupakaranant, P.; Chemat, F. Extraction of α-mangostin from *Garcinia mangostana* L. using alternative solvents: Computational predictive and experimental studies. *LWT*, **2016**, *65*, 297-303.
9. Filly, A.; Fabiano-Tixier, A. S.; Lemasson, Y.; Roy, C.; Fernandez, X.; Chemat, F. Extraction of aroma compounds in blackcurrant buds by alternative solvents: Theoretical and experimental solubility study. *C.R. Chim.*, **2014**, *17(12)*, 1268-1275.
10. Ballesteros-Vivas, D.; Álvarez-Rivera, G.; Morantes, S. J.; del Pilar Sánchez-Camargo, A.; Ibáñez, E.; Parada-Alfonso, F.; Cifuentes, A. An integrated approach for the valorization of mango seed kernel: Efficient extraction solvent selection, phytochemical profiling and antiproliferative activity assessment. *Food Res. Int.*, **2019**, *126*, 108616.
